# Breakfast habits, sedentary behavior, and suicide among Korean adolescents: A cross-sectional national study

**DOI:** 10.1371/journal.pone.0285312

**Published:** 2023-05-04

**Authors:** Ji-Su Kim, Yeji Seo

**Affiliations:** 1 Department of Nursing, Chung-Ang University, Seoul, Republic of Korea; 2 Department of Nursing, Semyung University, Chungbuk, Republic of Korea; Xi’an Jiaotong University, CHINA

## Abstract

This study aims to identify the relationships between breakfast habits, leisure-time sedentary behavior, and suicidal behaviors among Korean adolescents, including the mediating effect of leisure-time sedentary behavior on the relationship between breakfast habits and suicidal behaviors. We conducted a cross-sectional national study using secondary data from the 2017–2019 (13–15th) Korea Youth Risk Behavior Web-Based Surveys, analyzing data from a final sample of 153,992 Korean adolescents using multivariate logistic regression. No breakfast habits were statistically significantly related to suicidal ideation (crude OR [COR], 95% CI = 1.218, 1.172–1.265), suicidal plans (COR, 95% CI = 1.305, 1.228–1.385), and suicide attempts (COR, 95% CI = 1.533, 1.432–1.642). The effects of breakfast habits (independent variable) on suicidal behaviors (outcome variables) were mediated by leisure-time sedentary behavior (mediating variable). Leisure-time sedentary behavior had a statistically significant indirect effect on breakfast habits and suicidal behaviors (*p* < 0.05). The mediating effect size of breakfast habits mediated by leisure-time sedentary behavior was 3.46% for suicidal ideation, 2.48% for suicidal plans, and 1.06% for suicide attempts. Adolescents who did not consume breakfast demonstrated a significantly higher possibility of suicidal ideation, suicidal plans, and suicide attempts. Parents and teachers should be aware of and monitor adolescents’ leisure-time sedentary behavior and breakfast habits to prevent suicidal behavior among this age group.

## Introduction

The rising rate of suicidal behaviors is a growing problem among adolescents. Adolescents’ suicidal behaviors are linked to physical disorders and can create social, economic, and psychological burdens on communities, families, and individuals [1]. Currently, suicide is the dominant cause of death around the world [2]. Among adolescents, non-fatal suicidal behaviors, which include having suicidal thoughts, plans, and attempts, are much more common than fatal suicide [3]. Exploring variables related to suicidal behaviors among adolescents is imperative to prevent suicide and develop targeted interventions. The rate of suicide in South Korea is higher than that of the Organization for Economic Co-operation and Development countries and rose to 9.1 per 100,000 in 2018 [4].

Depression and pressure related to academic performance are significant causes of adolescent suicide and leisure-time sedentary behavior has recently been linked with suicidal behaviors [1]. Sedentary behavior refers to behavior involving less than 1.5 metabolic equivalents of energy, such as sitting or lying back [5], and is related to an increase in psychological distress and mental disorders [6]. Increasing sedentary behavior has a deleterious effect on adolescents and is linked to the levels of c-reactive protein and interleukin-6, both related to suicidal behaviors [7].

Daily breakfast also showed a dose effect on preventing adolescent suicide attempts [8]. Breakfast habits during adolescence affects lifelong health, including physical, psychological, and emotional development [9]. Adolescence is a time of rapidly developing physical and emotional health, and adolescents’ breakfast habits are associated with suicidal behaviors, including poor concentration and depression [10]. According to one study, only 63% of adolescents consumed regular breakfast [11]. In Korea, skipping breakfast is a growing trend due to social structure and related change [12], and it is necessary to look closely at adolescents’ breakfast habits in relation to suicidal behaviors.

Irregular breakfast habits lead to negative consequences, including leisure-time sedentary behavior and suicidal behaviors among adolescents [9]. Skipping breakfast leads to fatigue and more sedentary behavior [13], such as long duration of watching television, internet usage, or physical inactivity, a risk factor for suicidality and chronic diseases among adolescents [14]. Leisure-time sedentary behavior involving a low exertion of energy is associated with adolescents’ mental health and suicidal behavior caused by skipping breakfast [9]. Academic-oriented cultural characteristics in Korea might make sedentary behavior stronger among Korean adolescents [15]. Although this is an important topic, there are few studies on adolescents’ suicidal behaviors considering the mediating role of leisure-time sedentary behavior and breakfast habits.

Previous studies have examined the relationship between breakfast habits, sedentary behavior and suicide among adolescents [1, 16]. however, there has been no study focusing on the mediating role of sedentary behavior. Thus, it seems reasonable to hypothesize that sedentary behavior might mediate the association between breakfast habits and suicide.

Moreover, this study specially analyzed cross-sectional data from the KYRBWS, a survey of middle- and high-school students, to understand the current status and trends of health behaviors, such as diet, smoking, drinking, physical activity, mental health of Korean adolescents. A sample of middle- and high-school students representing the whole country was obtained using stratified multi-stage sampling, and students were surveyed anonymously during regular class time based on a self-filling web [17].

Therefore, this study aimed to identify the relationships between breakfast habits, leisure-time sedentary behavior, and suicidal behaviors among Korean adolescents and explore the mediating effect of leisure-time sedentary behavior. The specific objectives were to 1) explore breakfast habits and leisure-time sedentary behavior by demographic characteristics, 2) investigate suicidal behaviors by demographic characteristics and leisure-time sedentary behavior, 3) investigate the relationship between breakfast habits and suicidal behaviors, and 4) explore the mediating effect of leisure-time sedentary behavior.

## Materials and methods

### Participants

This study aimed to identify the relationships between breakfast habits, leisure-time sedentary behavior, and suicidal behaviors among Korean adolescents, including the mediating effect of leisure-time sedentary behavior on the relationship between breakfast habits and suicidal behaviors. We conducted a cross-sectional study using secondary data from the 2017–2019 (13–15th) Korea Youth Risk Behavior Web-Based Survey (KYRBWS). The study was approved by the Institutional Review Board (IRB) of the Korea Centers for Disease Control and Prevention (KCDC) in South Korea [17]. The KYRBWS is a self-administered online survey conducted with adolescents aged 12–18 years for exploring health behavior. Each adolescent’s parents provided written consent for the survey. Participating students were each given an identification number by their homeroom teachers, and they used these numbers to anonymously log into the web site and fill out the online questionnaire.

We used a multistage stratified cluster sampling design, selecting 400 participants according to stratification variables of school grade and city size. Sampling weights were calculated to analyze and minimize bias of selection according to the guidelines of the KYRBWS.

We analyzed the final data derived from 153,992 adolescents in the 13th–15th KYRBWS. The 2017 (13th) KYRBWS had 62,276 participants (response rate: 95.8%), the 2018 (14th) survey had 60,040 (response rate: 95.6%), and the 2019 (15th) survey had 57,303 (response rate: 95.3%) [18–20].

### Ethic consideration

This study was exempt from ethical approval by the IRB of Chung-Ang University (IRB No. 1041078-202008-HRSB-223-01). The datasets supporting the conclusions of this article are publicly available in the Korea Youth Risk Behavior Web-Based Survey (KYRBWS), at http://www.kdca.go.kr/yhs/.

### Measures

#### Breakfast habits

Breakfast habits were measured with the question “In the last 7 days, how many days did you eat breakfast (except milk or juice only)?” with answer options: 0, 1, 2, 3, 4, 5, 6, or 7 days. In the survey, bread, sunsik, or a powder made of mixed grains, porridge, and cereal were included within eating breakfast. The response for this question was dichotomized as “yes (at least once)” or “no (none).”

Especially, bread, seonsik, rice flour, porridge, cereal, etc. are included in the meal. In this question, it was reflected as a question that considered to some extent the case of eating food equivalent to breakfast, even if it was a small amount.

#### Leisure-time sedentary behavior

Leisure-time sedentary behavior was measured with the question “How much time do you spend during a typical or usual day sitting and watching television, playing computer games, surfing the internet, talking with friends, or engaged in other sitting activities on a weekday?” This variable included only weekday time, excluding time at school, and doing homework. This response was measured in minutes and was categorized as a dichotomized variable “yes (≥180 minutes/day)” or “no (<180 minutes/day)” [21].

#### Suicidal behaviors

Suicidal behaviors were measured by the following questions: “During the last 12 months, have you seriously considered suicide?” (suicidal ideation); “During the past 12 months, have you made specific plans to commit suicide?” (suicidal plans); and “During the last 12 months, how many times did you actually attempt suicide?” (suicide attempts). Each response was measured as a “yes” or “no.”

### Demographic characteristics

Demographic characteristics included age (years), gender (male, female), school grade (middle school, high school), city scale (metropolis, medium city, country), economic status (high, high-middle, middle, middle-low, low), school performance (high-middle, middle, middle-low, low), educational level of father and mother (middle school, high school, college or over, unknown, not living with), perception of depression, and whether the respondents had ever consumed alcohol, smoked, or used drugs.

All responses for the perception of depression and having consumed alcohol, smoked, or used drugs were dichotomized as “yes” or “no.”

Consumption of fast food was determined by the question “During the past 7 days, on how many days did you eat fast food?” with specific fast food examples. The answer for this question was “none,” “1–2 times per week,” “3–4 times per week,” “5–6 times per week,” “once per day,” “twice per day,” “three or more times per day.” This response was dichotomized as “yes (at least once)” or “no (none).” Consumption of carbonated drink was assessed through the question “In the last 7 days, how often did you drink soda (except carbonated water)?” This response was dichotomized as “yes (at least once)” or “no (none).”

### Data analysis

Data were analyzed using IBM SPSS version 25.0 (SPSS Inc., Chicago, IL, USA) considering a complex sample design. Sampling weights were adjusted according to the guideline of the KYRBWS. Descriptive statistics showed the mean ± standard error for continuous variables and the percentages (standard error) for categorical variables. Chi-square tests were used to analyze differences in breakfast habits and suicidal behaviors by demographic characteristics and leisure-time sedentary behavior for categorical variables. The generalized linear model was used considering sampling weights required in complex sample survey data for continuous variables by Lewis [22]. It was employed to analyze the differences of age and leisure-time sedentary behavior (in minutes) as continuous variables between each group of breakfast habits, suicidal behaviors, and leisure-time sedentary behavior. The expected marginal mean of age and leisure-time sedentary behavior were estimated in each group.

We analyzed the relationship between breakfast habits and suicidal behaviors. The multivariate logistic regression model was analyzed, adjusting for age, gender, school grade, city scale, economic status, school performance, education level of father and mother, perception of depression, alcohol consumption, smoking, drug use, consumption of fast food, and carbonated drinks according to previous studies [23, 24]. We presented odds ratios (ORs) and confidence intervals (95% CIs) adjusting multivariate variables. A *p*-value < 0.05 was considered statistically significant.

We also identified the mediating effect of leisure-time sedentary behavior on the relationship between breakfast habits and suicidal behaviors by applying the model developed by Baron and Kenny [25], which proposed estimators of indirect and direct effects of the independent variable on an outcome variable. Alwin & Hauser’s path analysis method and Baron & Kenny’s method for testing mediating effects are traditionally the most used and theoretically verified methods [26]. Some parts of the indirect effects are mediated by a covariate, and some of the direct effects are not. In this study, the outcome variable was suicidal behaviors, the independent variable was breakfast habits, and the mediating variable was leisure-time sedentary behavior. According to Baron and Kenny’s [25] model, mediation through leisure-time sedentary behavior is the indirect effect of breakfast habits on suicidal behaviors.

Finally, we statistically examined the indirect effect of leisure-time sedentary behavior on the relationship between breakfast habits and suicidal behaviors using the Sobel test [27]. We identified the mediation effect size according to Alwin and Hauser [28], who stated that the total effects mean the direct and the indirect effects, wherein the latter is equal to those influences mediated by the mediating variable. The mediating effect can be represented by the indirect effect to total effect ratio.

## Results

### Breakfast habits and leisure-time sedentary behavior by demographic characteristics

Table 1 represents statistical significance in breakfast habits by demographic characteristics. The highest rate of breakfast habits was found in the following groups: female; metropolis or country; high or high-middle economic status; high, high-middle, or middle school performance; college or above in educational level of father and mother; no perception of depression; those who did not consume alcohol, smoke, or use drugs; consumption of fast food; no leisure-time sedentary behavior (<180 minutes/day); shorter leisure-time sedentary behavior in minutes.

**Table 1:** Breakfast habits and leisure-time sedentary behaviors by demographic characteristics (N = 153,992).

Variable	Classification	Total Mean or % ± (SE)	Breakfast habits Mean or % ± (SE)		*p*	Leisure-time sedentary behavior Mean or % ± (SE)		*p*
			No (n = 30,317)	Yes (n = 123,675)		No (<3 hours/day) (n = 89,001)	Yes (≥3 hours/day) (n = 64,991)	
Age (years)		15.10±0.014	15.12±0.017	15.10±0.014	0.092	15.14±0.015	15.05±0.014	<0.001
Gender (%)	Male	50.6(0.7)	51.3(0.8)	50.5(0.8)	0.019	52.4(0.8)	48.2(0.7)	<0.001
	Female	49.4(0.7)	48.7(0.8)	49.5(0.8)		47.6(0.8)	51.8(0.7)	
School grade (%)	Middle school	47.1(0.4)	52.9(0.5)	52.8(0.5)	0.833	45.1(0.5)	50.0(0.5)	<0.001
	High school	52.9(0.4)	47.1(0.5)	47.2(0.5)		54.9(0.5)	50.0(0.5)	
City scale (%)	Metropolis	42.8(0.4)	42.2(0.5)	42.9(0.4)	0.035	43.1(0.5)	42.4(0.4)	0.108
	Medium city	51.2(0.5)	52.0(0.6)	51.0(0.5)		50.8(0.5)	51.7(0.5)	
	Country	6.0(0.3)	5.8(0.3)	6.1(0.3)		6.1(0.3)	5.9(0.3)	
Economic status (%)	High	10.7(0.1)	10.4(0.2)	10.7(0.1)	<0.001	11.6(0.1)	9.5(0.1)	<0.001
	High-middle	29.7(0.2)	26.3(0.3)	30.4(0.2)		31.1(0.2)	27.7(0.2)	
	Middle	46.3(0.2)	47.4(0.3)	46.1(0.2)		45.2(0.2)	47.9(0.2)	
	Middle-low	11.1(0.1)	12.7(0.2)	10.7(0.1)		10.2(0.1)	12.3(0.1)	
	Low	2.2(0.0)	3.1(0.1)	2.1(0.0)		1.9(0.0)	2.7(0.1)	
School performance (%)	High	13.2(0.1)	10.2(0.2)	13.9(0.1)	<0.001	15.2(0.1)	10.4(0.1)	<0.001
	High-middle	25.6(0.1)	20.9(0.2)	26.7(0.1)		27.7(0.2)	22.8(0.2)	
	Middle	29.3(0.1)	28.3(0.3)	29.6(0.1)		29.4(0.2)	29.2(0.2)	
	Middle-low	22.2(0.1)	26.4(0.3)	21.2(0.1)		19.9(0.1)	25.3(0.2)	
	Low	9.7(0.1)	14.2(0.2)	8.6(0.1)		7.8(0.1)	12.3(0.1)	
Educational level of father	Middle school	1.6(0.0)	1.8(0.1)	1.5(0.0)	<0.001	1.4(0.0)	1.8(0.1)	<0.001
	High school	24.0(0.2)	26.2(0.3)	23.5(0.2)		23.2(0.2)	25.2(0.2)	
	College or over	53.4(0.3)	46.2(0.4)	55.0(0.3)		56.1(0.4)	49.6(0.4)	
	Unknown	17.2(0.2)	20.7(0.3)	16.4(0.2)		15.8(0.2)	19.0(0.2)	
	Not living with	3.8(0.1)	5.1(0.1)	3.6(0.1)		3.4(0.1)	4.4(0.1)	
Educational level of mother	Middle school	1.4(0.0)	1.6(0.1)	1.3(0.0)	<0.001	1.3(0.0)	1.5(0.1)	<0.001
	High school	29.4(0.2)	31.8(0.3)	28.8(0.2)		28.5(0.3)	30.6(0.3)	
	College or over	49.8(0.3)	42.5(0.4)	51.5(0.3)		52.3(0.3)	46.4(0.4)	
	Unknown	16.3(0.1)	19.8(0.3)	15.5(0.1)		15.1(0.2)	17.9(0.2)	
	Not living with	3.2(0.1)	4.3(0.1)	3.0(0.1)		2.9(0.1)	3.6(0.1)	
Perception of depression (%)	Yes	26.4(0.2)	29.3(0.3)	25.7(0.2)	<0.001	24.9(0.2)	28.5(0.2)	<0.001
	No	73.6(0.2)	70.7(0.3)	74.3(0.2)		75.1(0.2)	71.5(0.2)	
Ever consumed alcohol (%)	Yes	40.4(0.2)	45.2(0.4)	39.3(0.2)	<0.001	38.7(0.3)	42.7(0.3)	<0.001
	No	59.6(0.2)	54.8(0.4)	60.7(0.2)		61.3(0.3)	57.3(0.3)	
Ever smoked (%)	Yes	13.5(0.2)	16.7(0.3)	12.8(0.2)	<0.001	12.5(0.2)	14.8(0.2)	<0.001
	No	86.5(0.2)	83.3(0.3)	87.2(0.2)		87.5(0.2)	85.2(0.2)	
Ever used drug (%)	Yes	0.8(0.0)	1.0(0.1)	0.8(0.0)	0.002	0.8(0.0)	0.9(0.0)	0.007
	No	99.2(0.0)	99.0(0.1)	99.2(0.0)		99.2(0.0)	99.1(0.0)	
Consumption of fast food (%)	Yes	81.7(0.1)	80.4(0.2)	80.9(0.1)	<0.001	79.2(0.2)	83.0(0.2)	<0.001
	No	18.3(0.1)	19.6(0.2)	19.1(0.1)		20.8(0.2)	17.0(0.2)	
Consumption of carbonated drink (%)	Yes	79.5(0.2)	81.1(0.3)	79.1(0.2)	0.071	77.7(0.2)	82.0(0.2)	<0.001
	No	20.5(0.2)	18.9(0.3)	20.9(0.2)		22.3(0.2)	18.0(0.2)	
Leisure-time sedentary behavior (%)	Yes(≥180 minutes/day)	41.9(0.2)	46.2(0.3)	41.0(0.2)	<0.001			
	No(<180 minutes/day)	58.1(0.2)	53.8(0.3)	59.0(0.2)				
Leisure-time sedentary behavior (minutes)		167.07±0.537	179.46±0.995	164.29±0.554	<0.001	82.68±0.177	283.92±0.687	<0.001

*Note*. SE = standard error.

Moreover, there were significant differences in leisure-time sedentary behavior among different demographic characteristics. This study revealed meaningful results in leisure-time sedentary behavior (≥180 minutes/day) as follows: younger; female; middle school; middle, middle-low, or low economic status; middle-low or low school performance; individuals whose father and mother did not complete college; perception of depression; those who consumed alcohol, smoked, or used drugs; consumption of fast food and carbonated drinks; higher leisure-time sedentary behavior.

### Suicidal behaviors by demographic characteristics and leisure-time sedentary behavior

Our study revealed significant results in suicidal ideation for the following groups (Table 2): younger; female; middle school; middle-low or low in economic status; middle school or not living with for educational level of father and mother; having a perception of depression; those who consumed alcohol, smoked, or used drugs; no breakfast habits; consumption of fast food; leisure-time sedentary behavior (≥180 minutes/day); and longer leisure-time sedentary behavior in minutes.

**Table 2:** Suicidal behaviors by demographic characteristics and leisure-time sedentary behaviors (N = 153,992).

Variable	Classification	Total Mean or % ± (SE)	Suicidal ideation Mean or % ± (SE)		*p*	Suicidal plans Mean or % ± (SE)		*p*	Suicide attempts Mean or % ± (SE)		*p*
			No (n = 134,269)	Yes (n = 19,723)		No (n = 147,827)	Yes (n = 6,165)		No (n = 149,703)	Yes (n = 4,289)	
Age (years)		15.10±0.014	15.11±0.014	15.06±0.019	0.009	15.11±0.014	14.93±0.026	<0.001	15.11±0.014	14.86±0.032	<0.001
Gender (%)	Male	50.6(0.7)	52.7(0.7)	36.7(0.8)	<0.001	51.1(0.7)	40.1(0.9)	<0.001	51.1(0.7)	34.3(0.9)	<0.001
	Female	49.4(0.7)	47.3(0.7)	63.3(0.8)		48.9(0.7)	59.9(0.9)		48.9(0.7)	65.7(0.9)	
School grade (%)	Middle school	47.1(0.4)	46.8(0.4)	49.8(0.6)	<0.001	46.8(0.4)	54.8(0.8)	<0.001	46.9(0.4)	56.5(1.0)	<0.001
	High school	52.9(0.4)	53.2(0.4)	50.2(0.6)		53.2(0.4)	45.2(0.8)		53.1(0.4)	43.5(1.0)	
City scale (%)	Metropolis	42.8(0.4)	42.8(0.4)	42.8(0.6)	0.423	42.8(0.4)	43.1(0.8)	<0.001	42.8(0.4)	41.8(0.9)	0.537
	Medium city	51.2(0.5)	51.2(0.5)	51.4(0.6)		51.2(0.5)	51.4(0.9)		51.2(0.5)	52.0(1.0)	
	Country	6.0(0.3)	6.1(0.3)	5.8(0.4)		6.0(0.3)	5.6(0.5)		6.0(0.3)	6.2(0.5)	
Economic status (%)	High	10.7(0.1)	10.9(0.1)	9.3(0.2)	<0.001	10.7(0.1)	11.4(0.4)	0.37	10.7(0.1)	10.8(0.5)	<0.001
	High-middle	29.7(0.2)	30.2(0.2)	26.4(0.3)		29.8(0.2)	25.7(0.6)		29.8(0.2)	24.8(0.7)	
	Middle	46.3(0.2)	46.9(0.2)	42.8(0.4)		46.6(0.2)	39.1(0.6)		46.5(0.2)	39.8(0.7)	
	Middle-low	11.1(0.1)	10.2(0.1)	16.7(0.3)		10.8(0.1)	17.2(0.5)		10.9(0.1)	18.0(0.6)	
	Low	2.2(0.0)	1.9(0.0)	4.8(0.2)		2.1(0.0)	6.6(0.3)		2.1(0.0)	6.6(0.4)	
School performance (%)	High	13.2(0.1)	13.4(0.1)	11.5(0.2)	<0.001	13.2(0.1)	12.1(0.4)	<0.001	13.3(0.1)	10.9(0.5)	<0.001
	High-middle	25.6(0.1)	26.1(0.1)	22.2(0.3)		25.8(0.1)	20.8(0.5)		25.8(0.1)	19.2(0.6)	
	Middle	29.3(0.1)	29.7(0.1)	26.8(0.3)		29.5(0.1)	25.8(0.6)		29.5(0.1)	25.0(0.7)	
	Middle-low	22.2(0.1)	21.7(0.1)	25.4(0.3)		22.1(0.1)	24.4(0.5)		22.1(0.1)	25.9(0.6)	
	Low	9.7(0.1)	9.0(0.1)	14.2(0.2)		9.4(0.1)	16.8(0.5)		9.4(0.1)	19.0(0.6)	
Educational level of father (%)	Middle school	1.6(0.0)	1.5(0.0)	2.0(0.1)	<0.001	1.6(0.0)	2.2(0.2)	<0.001	1.6(0.0)	2.3(0.2)	<0.001
	High school	24.0(0.2)	24.0(0.2)	24.0(0.3)		24.1(0.2)	22.7(0.5)		24.0(0.2)	24.2(0.7)	
	College or over	53.4(0.3)	53.4(0.3)	53.0(0.5)		53.4(0.3)	52.3(0.7)		53.5(0.3)	48.1(0.8)	
	Unknown	17.2(0.2)	17.4(0.2)	15.7(0.3)		17.2(0.2)	16.8(0.5)		17.1(0.2)	18.1(0.6)	
	Not living with	3.8(0.1)	3.6(0.1)	5.3(0.2)		3.8(0.1)	5.9(0.3)		3.7(0.1)	7.2(0.4)	
Educational level of mother (%)	Middle school	1.4(0.0)	1.3(0.0)	1.8(0.1)	<0.001	1.3(0.0)	1.8(0.2)	<0.001	1.3(0.0)	2.0(0.2)	<0.001
	High school	29.4(0.2)	29.4(0.2)	29.3(0.4)		29.4(0.2)	27.9(0.6)		29.3(0.2)	29.8(0.7)	
	College or over	49.8(0.3)	49.8(0.3)	49.9(0.5)		49.8(0.3)	49.5(0.7)		50.0(0.3)	44.4(0.8)	
	Unknown	16.3(0.1)	16.5(0.1)	14.5(0.3)		16.3(0.1)	15.3(0.5)		16.2(0.1)	17.1(0.6)	
	Not living with	3.2(0.1)	3.0(0.1)	4.5(0.1)		3.1(0.1)	5.5(0.3)		3.1(0.1)	6.7(0.4)	
Perception of depression (%)	Yes	26.4(0.2)	19.4(0.1)	74.4(0.3)	<0.001	24.3(0.1)	78.2(0.5)	<0.001	24.9(0.2)	79.6(0.6)	<0.001
	No	73.6(0.2)	80.6(0.1)	25.6(0.3)		75.7(0.1)	21.8(0.5)		75.1(0.2)	20.4(0.6)	
Ever consumed alcohol (%)	Yes	40.4(0.2)	38.7(0.2)	51.8(0.4)	<0.001	39.8(0.2)	55.0(0.7)	<0.001	39.9(0.2)	59.0(0.8)	<0.001
	No	59.6(0.2)	61.3(0.2)	48.2(0.4)		60.2(0.2)	45.0(0.7)		60.1(0.2)	41.0(0.8)	
Ever smoked (%)	Yes	13.5(0.2)	12.6(0.2)	19.4(0.3)	<0.001	13.1(0.2)	23.2(0.6)	<0.001	13.1(0.2)	27.3(0.7)	<0.001
	No	86.5(0.2)	87.4(0.2)	80.6(0.3)		86.9(0.2)	76.8(0.6)		86.9(0.2)	72.7(0.7)	
Ever used drug (%)	Yes	0.8(0.0)	0.6(0.0)	2.6(0.1)	<0.001	0.6(0.0)	5.8(0.3)	<0.001	0.7(0.0)	6.7(0.4)	<0.001
	No	99.2(0.0)	99.4(0.0)	97.4(0.1)		99.4(0.0)	94.2(0.3)		99.3(0.0)	93.3(0.4)	
Breakfast habits (%)	Yes	81.7(0.1)	82.1(0.1)	79.0(0.3)	<0.001	81.8(0.1)	77.5(0.5)	<0.001	81.9(0.1)	74.6(0.7)	<0.001
	No	18.3(0.1)	17.9(0.1)	21.0(0.3)		18.2(0.1)	22.5(0.5)		18.1(0.1)	25.4(0.7)	
Consumption of fast food (%)	Yes	80.8(0.1)	80.7(0.1)	81.3(0.3)	0.048	80.8(0.1)	80.4(0.5)	0.471	80.8(0.1)	80.3(0.6)	0.393
	No	19.2(0.1)	19.3(0.1)	18.7(0.3)		19.2(0.1)	19.6(0.5)		19.2(0.1)	19.7(0.6)	
Consumption of carbonated drink (%)	Yes	79.5(0.2)	79.5(0.2)	79.4(0.3)	0.651	79.5(0.2)	79.0(0.5)	0.32	79.5(0.2)	79.6(0.6)	0.884
	No	20.5(0.2)	20.5(0.2)	20.6(0.3)		20.5(0.2)	21.0(0.5)		20.5(0.2)	20.4(0.6)	
Leisure-time sedentary behavior (%)	Yes(≥180 minutes/day)	41.9(0.2)	41.2(0.2)	46.7(0.4)	<0.001	41.7(0.2)	47.9(0.7)	<0.001	41.7(0.2)	49.1(0.8)	<0.001
	No(<180 minutes/day)	58.1(0.2)	58.8(0.2)	53.3(0.4)		58.3(0.2)	52.1(0.7)		58.3(0.2)	50.9(0.8)	
Leisure-time sedentary behavior (minutes)		167.07±0	165.10±0	180.51±1	<0.00	166.26±0	186.76±2	<0.00	166.32±0	193.85±2	<0.001
		.537	.555	.138	1	.538	.140	1	.538	.680	

*Note*. SE = standard error.

Regarding suicidal plans, this study revealed significant differences as follows: younger; female; middle school; metropolis or medium city in city scale; middle-low or low in school performance; middle school or not living with for educational level of father and mother; perception of depression; those who consumed alcohol, smoked, or used drugs; no breakfast habits; leisure-time sedentary behavior (≥180 minutes/day); higher leisure-time sedentary behavior in minutes.

Regarding suicide attempts, the analysis revealed meaningful differences as follows: younger; female; middle school; high or middle-low or low in economic status; middle-low or low in school performance; not college or higher educational level of father and mother; perception of depression; those who consumed alcohol, smoked, or used drugs; no breakfast habits; leisure-time sedentary behavior (≥180 minutes/day); and higher leisure-time sedentary behavior in minutes.

### Analysis of mediating effect of leisure-time sedentary behavior between breakfast habits and suicidal behaviors

Detailed results are presented in Table 3. There are regression coefficients of multivariate logistic regression between pathway A (Breakfast habits → Leisure-time sedentary behavior), pathway B (Leisure-time sedentary behavior → Suicidal behaviors), and pathway C (Breakfast habits → Suicidal behaviors). All regression coefficients of pathways A, B, and C were significant.

**Table 3:** Mediating effect of leisure-time sedentary behavior between breakfast habits and suicidal behaviors (N = 153,992).

Variables	Pathway A		Pathway B		Pathway C				Sobel test (*p*)	
	*Breakfast habits → Leisure-time sedentary behavior*		*Leisure-time sedentary behavior → Suicidal behaviors*		*Breakfast habits → Suicidal behaviors*					Ratio of the indirect effect to the total effect
	OR (95% CI)	*p*	OR (95% CI)	*p*	OR- (95% CI)	*p*	OR+ (95% CI)	*p*		
*Leisure-time sedentary behavior*	1.138(1.108–1.169)	<0.001								
Suicidal ideation			1.069(1.033–1.107)	<0.001	1.071(1.026–1.117)	0.027	1.069(1.024–1.115)	0.002	<0.001	0.0346
Suicidal plans			1.080(1.022–1.142)	0.006	1.115(1.045–1.189)	0.001	1.112(1.043–1.187)	0.001	0.008	0.0248
Suicide attempts			1.073(1.006–1.145)	0.032	1.266(1.176–1.363)	<0.001	1.264(1.174–1.360)	<0.001	0.036	0.0106

*Note*. OR = odds ratio, CI = confidence interval, OR- = unadjusted for leisure-time sedentary behavior, OR+ = adjusted for leisure-time sedentary behavior, Covariate: Age, Gender, School type, City type, Economic level, Academic performance, Educational level(Father, Mother), Perceived depression, Ever drank alcohol, Ever smoked, Ever used drugs, Fastfood consumption, Carbonated soft drink consumption

The effects of breakfast habits (independent variable) on suicidal behaviors (outcome variables) were mediated by leisure-time sedentary behavior (mediating variable). It is the indirect effect of breakfast habits on suicidal behaviors. The Sobel test showed that mediating effect of leisure-time sedentary behavior between breakfast habits and suicidal behaviors was statistically significant (all *p* < 0.05).

The indirect effect to total effects ratio (Table 3) indicates the mediating effect size, based on Alwin and Hauser [28]. This ratio is mediated by leisure-time sedentary behavior and the total effect of breakfast habits on suicidal behaviors. The effect size of breakfast habits mediated by leisure-time sedentary behavior was 3.46% for suicidal ideation, 2.48% for suicidal plans, and 1.06% for suicide attempts.

## Discussion

The results show that adolescents whose fathers or mothers have an educational qualification of college or higher are more likely to consume breakfast. In addition, adolescents who consume breakfast had a middle or higher rating in school performance. Korean adolescents who do not eat breakfast are at higher risk of leisure-time sedentary behavior than those who do and are likely to have suicidal behaviors, such as suicidal ideation and attempts. Moreover, Korean adolescents engaging in leisure-time sedentary behavior (≥180 minutes/day) are likely to have suicidal behaviors, correlated with longer leisure-time sedentary behavior.

In contrast to previous studies, which mainly focused on the relationship between sedentary behavior and suicide among adolescents [1, 16], the results of this study focused on the mediating effect of sedentary behavior on the relationship between breakfast habits and suicide.

Our mediation analysis revealed that Korean adolescents’ leisure-time sedentary behavior might be indirectly related to suicidal behaviors by breakfast habits, and it has a mediating effect on the association between breakfast habits and suicidal behaviors. Ultimately, we found that not consuming breakfast is correlated with a higher risk for leisure-time sedentary behavior, which is related to increased reports of suicidal behaviors. As in previous studies, keeping with the academic-oriented passive lifestyle of Korean adolescents, we found that the proportion of physical activity was relatively insufficient. Regarding the size of the mediating effects, leisure-time sedentary behavior had the greatest effect on the relationship between breakfast habits and suicidal ideation.

Although the adverse effects of breakfast habits and sedentary behavior on suicide have been confirmed by several studies [16, 29], the specific mechanisms driving this relationship are still unclear and remain to be investigated. Hence, it is important to identify mediators driving the relationship between breakfast habits and suicide to better understand the underlying processes. In general, adolescent sedentary behavior, characterized by activities such as watching television, using mobile phones, and reading, using computers, taking online classes, and playing games, is regarded as an influencing factor on suicide [30]. According to previous study, skipping daily breakfast has been linked to sedentary behaviors such as increased screen time [31]. Thus, it seems reasonable that sedentary behavior might mediate the association between breakfast habits and suicide.

This study aimed to identify the associations between adolescents’ breakfast habits, leisure-time sedentary behavior, and suicidal behaviors using mediation analysis of a nationally complex representative sample. Parents and teachers need to identify the status of breakfast habits among adolescents in helping prevent suicidal behavior. It is also necessary to encourage adolescents to have a balanced breakfast and to monitor their leisure-time sedentary behavior, such as viewing television, accessing the internet, or physical inactivity while sitting during breakfast. Teachers and school administrators should develop tailored health education programs focused on breakfast and sedentary behavior to prevent suicidal behavior.

Regular breakfast habits are significantly positively related to a high parental educational level [32], which positively influences school grades and academic achievement [33] and has a significant effect on the cognitive function within 4 hours post-ingestion [34]. Breakfast is one of the most critical meals of the day—contributing to about 20–35% of total daily energy, providing nutrient intake for nutritional balance among adolescents [35]. Skipping breakfast is associated with suicidal behavior, including mental distress and a depressive mood [36]. Low frequency of breakfast habits among adolescents is correlated with lower physical activity and greater sedentary behavior [37], and an increased risk of suicidal plans and attempts, and depression [9, 38]. Adolescents with sedentary behavior of over 8 hours per day are at a much higher risk of attempting suicide compared to those with less than 1 hour of sedentary behavior [1]. Uddin et al. [39] emphasized that both male and female adolescents with higher leisure-time sedentary behavior reported greater suicidal ideation, plans, and attempts than those with lower sedentary behavior.

The relationship between breakfast habits and suicidal behavior is an issue of global interest, possibly because of the increasing habit of skipping breakfast and the low quality of breakfast among adolescents in modern society [40]. In Korea, adolescents often skip breakfast to accommodate their busy academic schedules, influencing suicidal behaviors [41]. Considering the academic-oriented socio-cultural characteristics, Korean adolescents spend plenty of time sitting in school or doing homework; thus, the consequences of leisure-time sedentary behavior on suicidal behaviors are a notable health problem [12]. Korean adolescents aged 12–18 years spend about 8 hours a day sitting, compared to an average sitting time of 7.5 hours per day among US adolescents [42, 43]. Excess leisure-time sedentary behavior among adolescents can lead to adverse physical and psychological health effects, such as the increased risk of cardio-metabolic disease, lower self-esteem, and suicidal risk. Varying socio-cultural attitudes toward sedentary behavior and environmental factors may account for the difference in leisure-time sedentary behavior across countries [1]. Although Korean adolescents have realistic limitations related to after-school activity and self-study, it is recommended that they engage in physical activity outside of school [15].

Globally, the rate of irregular breakfast habits ranges from 27–62% among adolescents [10]. The rate of skipping breakfast among Korean adolescents is quite high at about 34%, likely due to changes in family structure (nuclear family) and an increase in women’s economic activity in modern society [44]. International reports of published physical activity guidelines in the United States, the United Kingdom, Australia, and Canada, have identified leisure-time sedentary behavior as a major modifiable variable related to adolescent health and emphasized that it should be minimized [45]. Among British adolescents as well, irregular breakfast habits were related to sedentary behavior, including lower physical activity [37].

Previous studies have explored the relationship between leisure-time sedentary behavior and suicidal ideation [41], but further investigation is required considering confounding factors for explaining mediating effect size.

Our study has several limitations. First, this study utilized a cross-sectional design; therefore, causal relationships must be interpreted with caution. Second, participants were asked only about the frequency of breakfast habits without considering the quality. Third, as our measurements were self-reported and administered, the data may suffer from response biases and low internal consistency. More objective and reliable measurements should be considered for the assessment of leisure-time sedentary behavior in the future.

## Conclusion

Parents and teachers need to be aware of the association between breakfast habits and suicidal behaviors and assess and monitor adolescents’ leisure-time sedentary behavior and breakfast habits to prevent suicidal behaviors among Korean adolescents. We recommend further research on educational interventions, including exercise and nutrition programs.

## Abbreviations

CI: confidence interval
COR: crude odds ratio
KCDC: Korea Centers for Disease Control and Prevention
KYRBWS: Korea Youth Risk Behavior Web-Based Survey
OR: odds ratio
